# Root Border Cells and Mucilage Secretions of Soybean, *Glycine Max* (Merr) L.: Characterization and Role in Interactions with the Oomycete *Phytophthora Parasitica*

**DOI:** 10.3390/cells9102215

**Published:** 2020-09-30

**Authors:** Marc Ropitaux, Sophie Bernard, Damien Schapman, Marie-Laure Follet-Gueye, Maïté Vicré, Isabelle Boulogne, Azeddine Driouich

**Affiliations:** 1 Laboratoire de Glycobiologie et Matrice Extracellulaire Végétale, UPRES-EA 4358, Fédération de Recherche « Normandie-Végétal »-FED 4277, Université de ROUEN Normandie, UFR des Sciences et Techniques, F-76821 Mont-Saint-Aignan, France; marc.ropitaux1@univ-rouen.fr (M.R.); sophie.bernard@univ-rouen.fr (S.B.); marie-laure.follet-gueye@univ-rouen.fr (M.-L.F.-G.); maite.vicre@univ-rouen.fr (M.V.); isabelle.boulogne@univ-rouen.fr (I.B.); 2 Cell Imaging Platform (PRIMACEN-IRIB), Université de ROUEN Normandie, UFR des Sciences et Techniques, F-76821 Mont-Saint-Aignan, France; damien.schapman@univ-rouen.fr

**Keywords:** border cells, bell wall, defense, ExDNA, heteromannan, immuno-microscopy, mucilage, polysaccharides, root, xyloglucan

## Abstract

Root border cells (BCs) and their associated secretions form a protective structure termed the root extracellular trap (RET) that plays a major role in root interactions with soil borne microorganisms. In this study, we investigated the release and morphology of BCs of *Glycine max* using light and cryo-scanning electron microscopy (SEM). We also examined the occurrence of cell-wall glycomolecules in BCs and secreted mucilage using immunofluorescence microscopy in conjunction with anti-glycan antibodies. Our data show that root tips released three populations of BCs defined as spherical, intermediate and elongated cells. The mechanism of shedding seemed to be cell morphotype-specific. The data also show that mucilage contained pectin, cellulose, extracellular DNA, histones and two hemicellulosic polysaccharides, xyloglucan and heteromannan. The latter has never been reported previously in any plant root secretions. Both hemicellulosic polysaccharides formed a dense fibrillary network embedding BCs and holding them together within the mucilage. Finally, we investigated the effect of the RET on the interactions of root with the pathogenic oomycete *Phytophthora parasitica* early during infection. Our findings reveal that the RET prevented zoospores from colonizing root tips by blocking their entry into root tissues and inducing their lysis.

## 1. Introduction

The root is a fascinating and dynamic organ that is critical to plant health and development. As it grows through the soil, the root tip of a plant is constantly exposed to a diversity of microbes and engaged in complex interactions within its surrounding rhizosphere environment [1,2]. The root tip produces a root cap whose primary function is to protect the meristem [3]. The root cap contains loosely attached peripheral cells that are programmed to separate from the root tissue and be released into the rhizosphere as viable and active secretory cells [4,5,6,7]. These cells are now widely known as border cells (BCs) or border-like cells (BLCs) [7,8,9,10,11,12,13]. Recently, Driouich et al. [14] proposed to group BCs and BLCs under the term root AC-DC (root Associated Cap-Derived Cells). The cells and their associated molecular secretions form an elaborate structure defined as the root extracellular trap (RET) and are recognized as a first line of defense against invading microbial pathogens [15,16]. The RET molecular complex consists of a variety of components including cell wall-derived polysaccharides, glycoproteins and various classes of anti-microbial compounds, critical to its function in biotic and abiotic interactions [14,15,16].

Many plant species produce root BCs [8,10] with the Leguminosae *Pisum sativum* L. (pea) being the most studied species to date, with regards to molecular composition and function of BCs and their secretions [7,8,9,16,17,18]. Immunocytochemical studies using a variety of anti-glycan antibodies have demonstrated the presence of different glycomolecules in the cell wall and mucilage of BCs of this species including pectins, xyloglucan, arabinogalactan proteins and extensins [12,17,18,19,20]. Many proteins were also identified in pea root BC secretions, including wall-modifying enzymes (e.g., xyloglucan endotransglycosylases), defense-related enzymes (e.g., chitinases and glucanases) and histone H4, a protein known to bind DNA and to inhibit bacterial growth [21,22,23,24]. In addition, and remarkably, extracellular DNA (exDNA) was also identified in the secretions of pea BCs and shown to function, along with histone H4, in preventing infection of root host tissues by the bacterium *Ralstonia solanacearum* (Smith) [24,25]. It is well established that pea root BCs and their secretions play a direct role in root protection against root-infecting pathogens, as demonstrated for the oomycete *Aphanomyces euteiches*–pea and the fungal pathogen *Nectria haematococc–*pea pathosystems [19,26]. In these studies, the entire structure formed by BCs and the mucilaginous secretions was shown to trap and repel fungal hyphae limiting pathogenic invasion of root tissues, whereas secreted arabinogalactan proteins were found to prevent zoospore germination and subsequent development of the oomycete *Aphanomyces euteiches* [17,19,26,27]. In both cases, neither pathogen was able to penetrate the root BCs. Interestingly, recent studies suggest that BCs and secretions (i.e., RETs) function in a manner similar to DNA-containing extracellular traps released by mammalian neutrophils in response to microbial infections [14,15,24,28,29,30]. Nonetheless, our understanding of the functional role of BCs and secreted compounds in other plant systems, including other important Legume crop species, remains limited.

In addition to their established role in biotic stress, root BCs from a range of species were also shown to protect root tips from abiotic stresses including soil physical abrasion [31,32] and heavy metal toxicity [33,34,35]. For instance, mucilage secretion was shown to increase significantly in response to Al in both green bean (*Beta vulgaris* L.) and soybean (*Glycine max* (Merr.) L.) plants [33,35]. In both species, root exposure to Al induced production of a thicker mucilage layer around BCs and root caps. In soybean, the secreted mucilage was shown to tightly bind the Al cations, thus preventing Al-induced damage of root tips, and removal of mucilage resulted in a severe arrest in root growth [35]. The authors suggested that the binding capacity of Al by the mucilage of soybean root BCs might be due to cell wall pectins that are possibly present in these secretions. However, whether these secretions also affect interactions between soybean roots and root-infecting microbes of the rhizosphere is currently unknown.

Soybean is a leguminous species similar to pea that is widely grown for its edible seeds for food and feed [36]. However, it is vulnerable to a variety of root-infecting pathogens that causes considerable losses of the crop worldwide [36,37,38,39]. Although this crop is as important as pea, very little is known regarding the molecular composition and function of root BCs and associated mucilage in relation to biotic stress [35].

Herein, we provide the first detailed characterization of root BCs and their secretions in *Glycine max*, an important crop member of the Leguminosae family using cell imaging and immunocytochemistry. The most important findings are: (i) soybean root tip released different BC types showing different morphologies, mucilage production and viability patterns; (ii) mucilage secretions consisted predominantly of cell wall polysaccharides, especially, heteromannan that has never been reported previously in root secretions; (iii) heteromannan and xyloglucan formed a highly dense structural network within the mucilage (i.e., a structural scaffold) possibly required for RET architecture and function; (iv) histone H4 protein and exDNA were also released into the mucilage; and (v) soybean RETs prevented *Phytophthora parasitica* zoospores from colonizing the root tip early during infection.

## 2. Materials and Methods

### 2.1. Plant Material

Soybean (*Glycine max* (L.) Merr.) seeds were sterilized overnight with chlorine gas. Briefly, seeds were placed in a beaker containing 100 mL of bleach in which 3 mL of concentrated HCl were added before being placed into a desiccator jar. After two washes with sterile water, soybean seeds were left out to imbibe at 22 °C in sterile water overnight. Seeds were then sown on Whatman paper soaked in half strength Murashige and Skoog medium at 22 °C for 5 days (16 h to 8 h day and night cycle).

### 2.2. Pathogen Culture and Inoculation of Soybean Seedlings

*P. parasitica* Dastur isolate 310 was kindly provided by Dr. Agnès Attard (Sophia Antipolis, INRAe, France). Mycelia were cultivated on 1% w/v V8-agar medium at 24 °C in the dark [40]. For zoospore production, mycelia were grown 1 week on V8 liquid medium at 24 °C under continuous light. After being washed 3 times using successive centrifugation at 1700× *g* and 25 °C, mycelia were incubated for a further 4 days in 2% w/v agar. Zoospores were released by heat shock treatment by first incubating at 4 °C for 30 min and then placed in water at 37 °C for 30 min. Zoospores were counted using a Nageotte cell to determine the number to inoculate with.

To obtain RET-less root tips, the RET was removed by gently rubbing root tips on Whatman paper. For observation, root tips with or without RETs were mounted on superfrost microscope slides, covered with an ultraviolet light-permeable cover slip. Then, 200 µL of water containing *P. parasitica* oospores (about 10^5^ zoospores/mL water) were added by capillarity. Root tips were observed for 15–30 min following inoculation using a bright-field microscope (Leica DMI6000B, Wetzlar, Germany). Observations were performed using three biological replicates with five technical replicates.

### 2.3. Light Microscopy

Roots were mounted on a microscope slide in a drop of water and examined directly for morphological analyses using a bright-field microscope (Leica DMI6000B, Wetzlar, Germany) and a stereomicroscope (Leica M125, Wetzlar, Germany). To examine the mucilage around BCs, India ink (Salis International Inc., Oceanside, CA, USA) staining was performed following a procedure adapted from Miyasaka and Hawes [33]. Briefly, mucilage was collected by dipping roots in a drop of water on a microscopic slide, and India ink was added by capillarity action between the slide and the cover slip. Root mucilage was observed using a bright-field microscope (Leica DMI6000B, Wetzlar, Germany). Mucilage from 30–35 roots was observed to ensure representativity for each set of observations.

### 2.4. Cryo-Scanning Electron Microscopy

Roots from five-day-old seedlings were fixed with 4% (w/v) paraformaldehyde (PFA; EMS, Hatfield, UK) in phosphate buffer saline (PBS 1×, pH 7). Roots were deposited onto a filter and fixed onto a stub using Tissue-Tek/colloidal graphite mounting media for subsequent freezing in slush nitrogen at −220 °C. The frozen sample was then transferred under vacuum to the cryo-chamber (Quorum PP3000T Cryo Transfer System, Puslinch, ON, Canada) cooled at −140 °C. Sublimation of the frozen specimen was performed at −95 °C for 20 min. Roots were then metalized with Platine (Pd) (60 s, 10 mA) and introduced into the microscope chamber where samples were maintained at −140 °C during observation, operating at 10 kV accelerating voltage. Cryo-SEM images were performed with a FEG FEI Quanta 250 microscope (FEI Company, Eindhoven, Holland) using a method adapted from Engström et al. [41].

### 2.5. Histochemical Staining

Cell viability was assessed with fluorescein diacetate (FDA) (Sigma-Aldrich, Saint-Quentin Fallavier, France) as previously described by Jones and Senft [42]. Roots were incubated with the fluorescent probe (0.5 mg·L^−1^) for 3 min and observed using an epifluorescence microscope (Leica DMI6000B, Wetzlar, Germany; λExcitation: 359 nm; λEmission: 461 nm).

Cytochemical staining of cellulose was carried out using Direct Red 23 (Sigma-Aldrich, Saint-Quentin Fallavier, France) as described previously [43]. Roots were incubated with the probe (0.1 mg·mL^−1^) for 30 min in darkness. After 3 washes with distilled water, roots were observed using a confocal laser-scanning microscope (Leica TCS SP5, Wetzlar, Germany; λExcitation: 560 nm; λEmission: 570–655 nm).

Staining of exDNA was performed as described by Wen et al. [44]. Sytox Green (Invitrogen, Carlsbad, CA, USA) solution was made by 1:1000 dilution in sterile water. Mucilage was released from root tips by contact with the surface of the glass slide, and 10 μL of diluted Sytox Green were added to the sample, which was then covered with a cover slip and observed using an epifluorescence microscope (Leica DMI6000B, Wetzlar, Germany; λExcitation: 359 nm; λEmission: 461 nm).

### 2.6. Immunofluorescence Labeling of Polysaccharide Epitopes and Histone H4

Details of the anti-cell wall polysaccharide and glycoprotein monoclonal antibodies (mAbs) (PlantProbes, Leeds, UK) used in this study are given in Table S1. The anti-histone H4 antibody was purchased from ThermoFischer (Breda, Holland). The secondary antibodies used were tetramethylrhodamine isothiocyanate (TRITC)-conjugated goat anti-rat (Sigma-Aldrich, Saint-Quentin Fallavier, France) and fluorescein isothiocyanate (FITC)-conjugated goat anti-mouse (Sigma-Aldrich, Saint-Quentin Fallavier, France).

Roots were placed onto sterile 18-welled Poly-l-Lysine microscope slides IBIDI (CliniSciences, Nanterre, France) and fixed for 40 min in 4% (w/v) PFA, in 50 mM PIPES (piperazine-N,N′-bis [2-ethanesulfonic acid]), pH 7, containing 1 mM CaCl_2_. Roots were washed 4 times at room temperature (RT) in PBS 1×, BSA 1% w/v (Bovine Serum Albumin; AURION, Wageningen, Holland) for 10 min each wash. Roots were then incubated overnight at 4 °C with the primary antibody (dilution 1:5 in 1× PBS containing 1% w/v BSA for all mAbs, except anti-histone H4 where a dilution of 1:10 was used). After 4 washes at RT with PBS 1× and 1% BSA for 10 min, roots were incubated with the secondary antibody (dilution 1:50 in PBS 1× and 1% BSA) for 2 h at 25 °C. Roots were rinsed 4 times at RT in PBS 1× containing 1% w/v BSA for 10 min, mounted in anti-fading solution (Citifluor AF2, Biedermannsdorf, Austria), and examined using a confocal laser-scanning microscope (Leica TCS SP5, Wetzlar, Germany; FITC = λExcitation: 494 nm; λEmission: 505–540 nm; TRITC = λExcitation: 550 nm; λEmission: 565–615 nm).

### 2.7. Xyloglucan and Histone H4 Double Immunolabeling

Root seedlings were placed onto a sterile 18-welled Poly-l-Lysine microscope slide IBIDI (CliniSciences, Nanterre, France) and fixed for 40 min in 4% PFA as described previously. Roots were washed 4 times at RT in PBS 1×, 1% BSA for 10 min each wash, and incubated overnight at 4 °C with the anti-mouse primary antibody (anti-histone H4; dilution 1:10 in PBS 1×, 1% BSA and normal goat serum 1/30). After 4 washes at RT with PBS 1× and 1% BSA for 10 min, roots were incubated with the secondary antibody coupled to FITC (dilution 1:75 in PBS 1× and 1% BSA) for 2 h at 25 °C. Roots were then rinsed 4 times at RT in PBS 1× and 1% BSA for 10 min. Then, samples were incubated with the anti-rat primary antibody (LM15 or LM25; dilution 1:5 in PBS and 1% BSA) overnight at 4 °C. After 4 washes at RT with PBS 1× and 1% BSA for 10 min, they were then incubated with the secondary antibody coupled to TRITC (dilution 1:75 in PBS 1× and 1% BSA) for 2 h at 25 °C, in a humidified chamber. After the last wash with PBS 1×, roots were mounted in anti-fading solution and examined using a confocal laser-scanning microscope (Leica TCS SP5, Wetzlar, Germany; FITC = λExcitation: 494 nm; λEmission: 505–540 nm; TRITC = λExcitation: 550 nm; λEmission: 565–615 nm).

### 2.8. Statistical and Image Analysis

XLSTAT 2018.1.1 software (Copyright Addinsoft 1995–2020) was used to statistically analyze the data. Statistical significance was calculated by using the Kruskal–Wallis test and the Mann–Whitney test, and *P* < 0.05 was accepted as significant. Microscope images were acquired and measurements made using Leica microscope systems software (LAS V3.8, Wetzlar, Germany) and Mesurim pro software ((J-F Madre, ENS, ACCES, Lyon, France). Stacks and stack deconvolutions were performed using ImageJ (V1.51) and Huygens Professional software (http://www.svi.nl).

## 3. Results

### 3.1. Cryo-Scanning Electron Microscopy Observation of Root BCs and Mucilage

**Figure 1:**
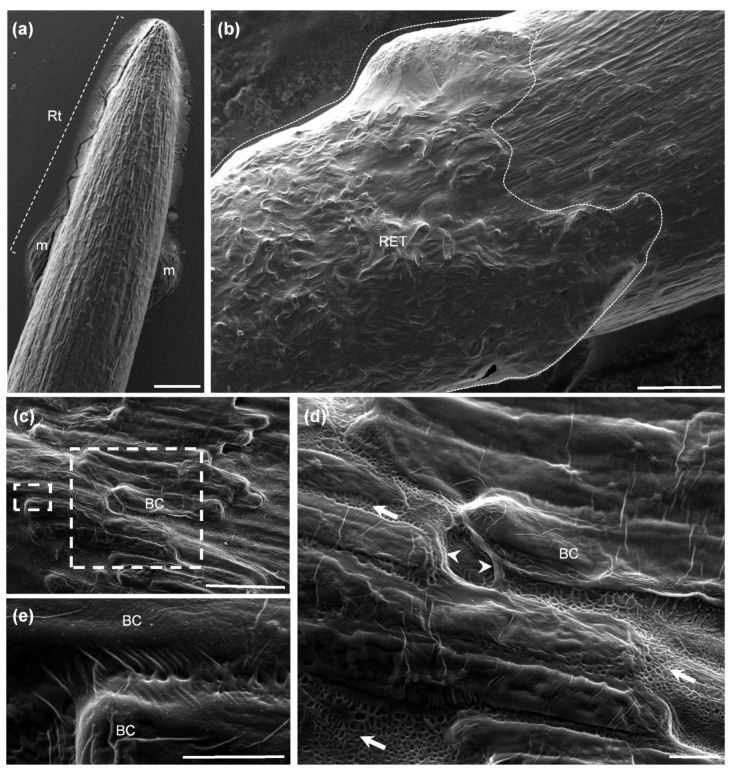
Morphology of a soybean root tip, border cells and mucilage observed by cryo-scanning electron microscopy. Micrographs showing a root tip surrounded by border cells and mucilage. (**a**–**c**) Border cells appear to be “glued” over the root surface and coated by dense extracellular material. (**d**,**e**) The material forms a fibrillary structure between the cells (white arrowheads), and fibrous elements are also observed probably linking cells together (white arrows). Boxed regions in (**c**) are shown at higher magnification in (**d**,**e**) to reveal details of the secreted mucilage. BC, border cells; m, mucilage; Rt, root tip; RET, Root Extracellular Trap. Scale bars: (**a**,**b**) 200 µm; (**c**) 100 µm; and (**d**,**e**) 10 µm.

Figure 1 shows cryo-scanning electron micrographs of a five-day-old soybean root tip. Image analysis revealed the presence of numerous BCs adhering tightly to the root tip surface (Figure 1a,b). Close examination of the surface revealed that the cells were embedded in a layer of extracellular material (i.e., mucilage) that seems to hold them together (Figure 1b,c). The material appeared as fibrous/fibrillary structures (white arrowheads) and formed a meshwork between the cells (white arrows) (Figure 1d). Bridge-like structures (threads) were also observed between the cells, possibly connecting them to each other (Figure 1e).

### 3.2. Different Morphotypes of BCs Are Released by the Root Tip

**Figure 2:**
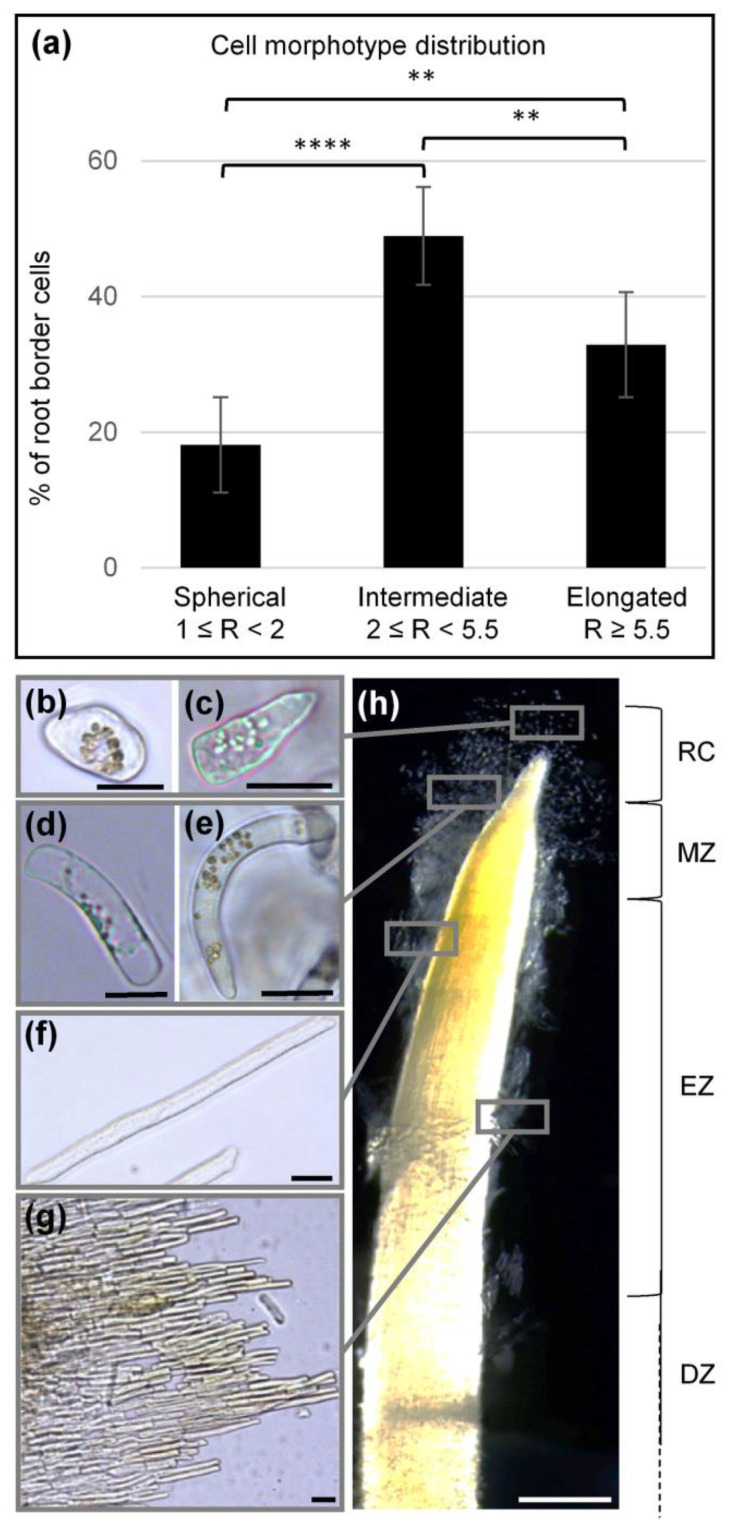
Root border cell morphotypes. (**a**) Histograms illustrating the proportion of different types of border cells. Three morphotypes were observed and defined as “spherical” (1 ≤ R < 2), “intermediate” (2 ≤ R < 5.5) and “elongated” (R ≥ 5.5). R is the cell length/cell width ratio. Each histogram represents the means of 12 biological replicates. Kruskal–Wallis multiple comparisons test used with Dunn’s correction. (**b**–**g**) Images showing different cell morphotypes using a bright-field microscope: (**b**) round or (**c**) slightly curved sBC; (**d**) rectangular or (**e**) curved iBC; and (**f**) isolated eBC; (**g**) eBC in the form of a stack containing several cells attached to each other. Image (**h**) illustrates the location of different cell types along the root tip with a stereomicroscope. Different root zones are: DZ, differentiation zone; EZ, elongation zone; MZ, meristematic zone; RC, root cap. Scale bars: (**b**,**c**) 20 µm; (**d**–**g**) 25 µm; and (**h**) 500 µm. ** *P* ≤ 0.01, **** *P* ≤ 0.0001

Careful observation of the root tip with a bright-field illumination revealed that BCs varied in size and shape (Figure 2). Calculation of cell length, cell width and the ratio of length to width (R) allowed three cellular morphotypes to be defined which we termed: spherical (1 ≤ R < 2), intermediate (2 ≤ R < 5.5) and elongated (R ≥ 5.5) BCs (Figure 2a). Measurements of cell lengths and widths are given in Table S2.

The proportion of each cell morphotype present within the root BC population is presented in Figure 2a. Spherical border cells (sBC) were the smallest and represented 18 ± 7% of the entire population. Types of sBC are shown in Figure 2b,c. Intermediate border cells (iBC) had a rectangular shape (Figure 2d,e) and accounted for 50 ± 7% of the entire BC population (Figure 2a). These cells displayed different degrees of curvature from moderate to nearly circular (See Figure 2d,e and Figure 8b). Elongated border cells (eBC) were 2–4.5 times longer than the iBC and sBC, displayed a rectangular shape (Figure 2f) and represented 33 ± 8% of the BC population (Figure 2a). Interestingly, eBC were observed either as single cells or as a group of several tightly attached cells that formed stacks (a dozen or a few dozen cells) (Figure 2g). The different cell morphotypes appeared to be localized to specific regions of the root tip (Figure 2h). sBC were mostly observed in the root cap zone, whereas iBC and eBC were found along the meristematic and the elongation/differentiation zones, respectively (Figure 2h).

### 3.3. Cell Viability and Mucilage Staining

**Figure 3:**
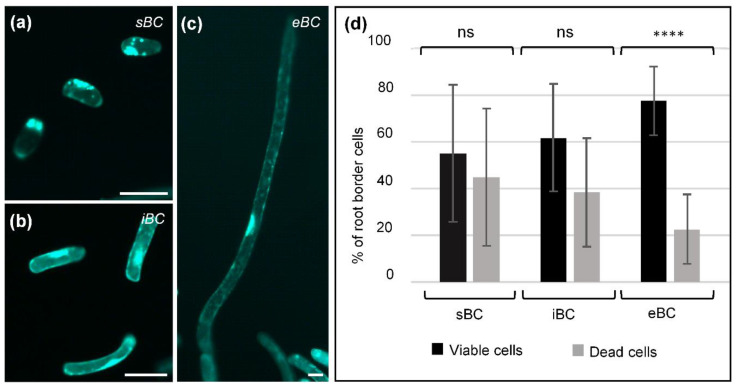
Cell viability. (**a**–**c**) Fluorescence images showing viable cells of the three BC morphotypes: (**a**) spherical; (**b**) intermediate; and (**c**) elongated. (**d**) Histograms represent quantitative data indicating the relative proportion of viable and non-viable cells for each morphotype. Each histogram represents the means of 12 biological replicates. (Mann–Whitney test used; ns, not significant; **** *P* ≤ 0.0001). Cell viability was assessed using Fluorescein diacetate (FDA) as described by Plancot et al. [45]. sBC, spherical border cells; iBC, intermediate border cells; eBC, elongated border cells. Scale bar: 50 µm.

Viability of the three BC morphotypes was assessed using Fluorescein diacetate (FDA) as described previously by Plancot et al. [45]. The presence of fluorescence was observed in the different cell populations, indicating that all BC morphotypes were viable (Figure 3a–c). However, the proportion of viable cells was different depending on the BC morphotype (Figure 3d and Table S2), with eBC presenting the highest percentage of viability (nearly 80%). In contrast, sBC morphotypes contained a relatively equal proportion of viable and dead cells. Bright field and fluorescence FDA images as well as negative controls are shown in Figure S1.

**Figure 4:**
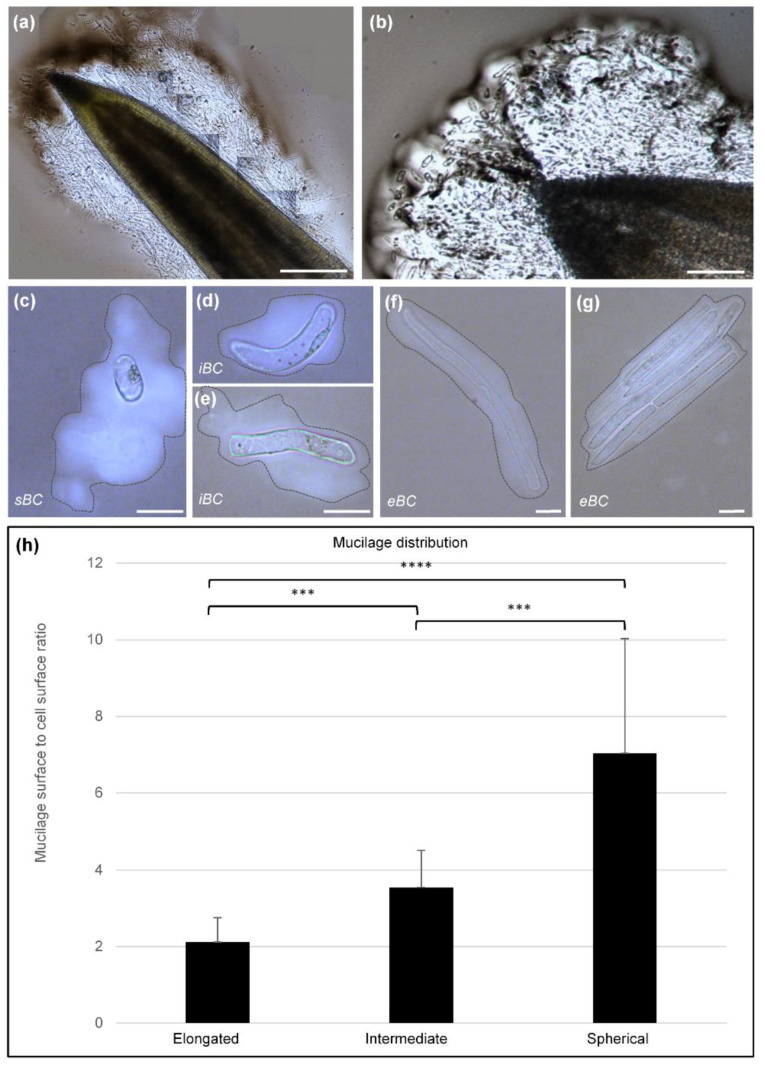
Visualization of secreted mucilage using India ink staining. (**a**,**b**) Light microscopy images showing an abundant slimy mucilage present around the root tip and embedding border cells. (**c**–**g**) images showing different cell types and their secreted mucilage (stained with India ink and delimited by a dashed line). (**h**) Histograms represent quantitative data indicating the proportion of mucilage surface surrounding each morphotype. R is the mucilage surface/cell surface ratio. Each histogram represents the ratio of 20 cells per morphotype, from 30 biological replicates. Kruskal–Wallis multiple comparisons test used with Bonferroni’s correction. sBC, spherical border cells; iBC, intermediate border cells; eBC, elongated border cells.; m, mucilage. Scale bars: (**a**) 300 µm; (**b**) 100 µm; and (**c**–**f**) 40 µm. *** *P* ≤ 0.001, **** *P* ≤ 0.0001

Negative staining of root tip with India ink revealed the presence of abundant mucilage secretions along the root tip (Figure 4a,b) and around isolated BC morphotypes (Figure 4c–g). The mucilage thickness and cell surface were measured using Mesurim software for each of the BC morphotypes (Figure 4h). The measured ratios revealed that sBC were surrounded by a thick layer of mucilage as compared to iBC and eBC. iBC produced less mucilage than sBC but much more of it than eBC. Interestingly, we observed that curved iBC exhibited polarized mucilage distribution (see Figure S2).

### 3.4. Molecular Characterization of the RET Released by Root Tips

To further characterize the RET (i.e., mucilage and BCs) released by soybean root tips, we performed specific staining and immunocytochemistry on BC wall and mucilage using light and confocal laser scanning microscopy. We used a set of specific monoclonal antibodies to assess the presence of glycan epitopes associated with cell wall polysaccharides and glycoproteins (Table S1). In addition, a specific probe that stains exDNA and an antibody specific for histone H4 protein were used. Figure S3 illustrates negative controls for immunolabeling experiments.

#### 3.4.1. Immunolocalization of Hemicellulosic Epitopes

**Figure 5:**
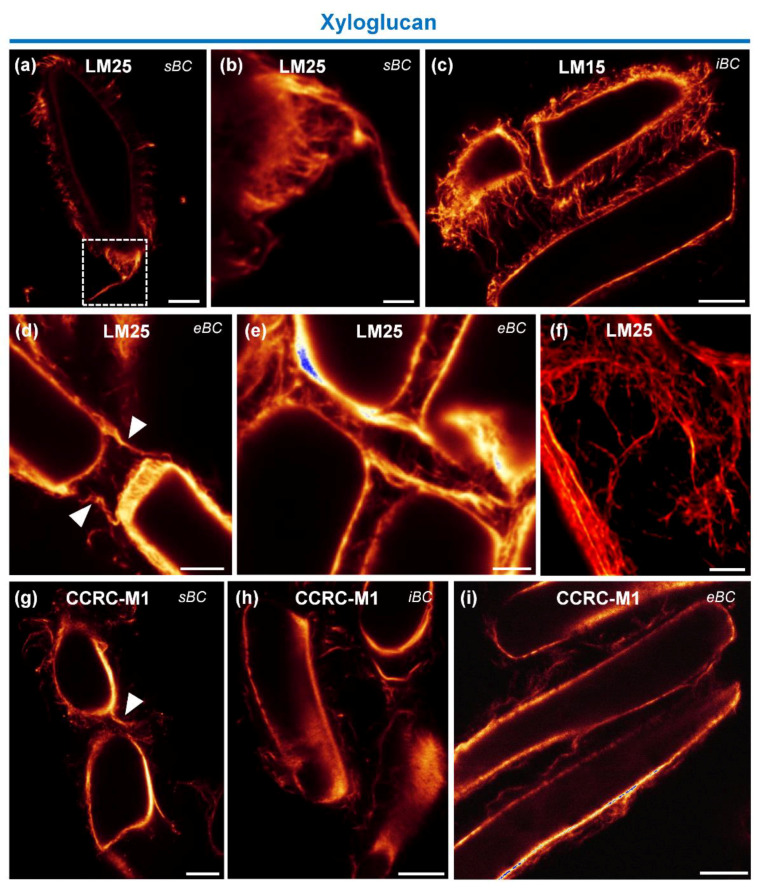
Immunolocalization of xyloglucan epitopes. (**a**–**h**) Immunofluorescence images showing labeling of the cell wall and extracellular material in all BC morphotypes with (**a**,**b**,**d**–**f**) the mAbs LM25; (**c**) LM15; and (**g**–**i**) CCRC-M1. Fluorescence labeling appears as a dense fibrous network surrounding the cells and sometimes connecting them together (arrowheads). (**a**–**c**) Fibrous structures appear to peel off the cell surface and form condensed threads. (**f**) Deconvolution analysis of samples labeled with LM25 in which the network appears even denser between cells. sBC, spherical border cells; iBC, intermediate border cells; eBC, elongated border cells. Scale bars: (**a**,**c**,**g**–**i**) 10 µm; (**d**) 7.5 µm; (**e**) 5 µm; and (**b**,**f**) 2.5 µm.

We investigated the occurrence of xyloglucan (XyG) using three mAbs, namely LM15, LM25 and CCRC-M1, which recognize xylosylated XyG, galactosylated XyG and fucosylated XyG, respectively [46,47,48,49]. As shown in Figure 5, all antibodies labeled the cell walls of all BC morphotypes, as well as the extracellular material surrounding these cells. The mAbs LM15 and LM25 labeled the mucilage more strongly than the mAb CCRC-M1 (Figure 5a–i and Figure S4). The fluorescence labeling obtained with the mAbs LM15 and LM25 appeared as a dense fibrous network surrounding the cells and sometimes showed strands connecting cells together (Figure 5c,d). In contrast, these same fibrous structures were faintly stained with the CCRC-M1 antibody (Figure 5g–i). Fluorescently labeled material between cells was also observed with the mAbs LM25 and CCRC-M1 (Figure 5d–f).

**Figure 6:**
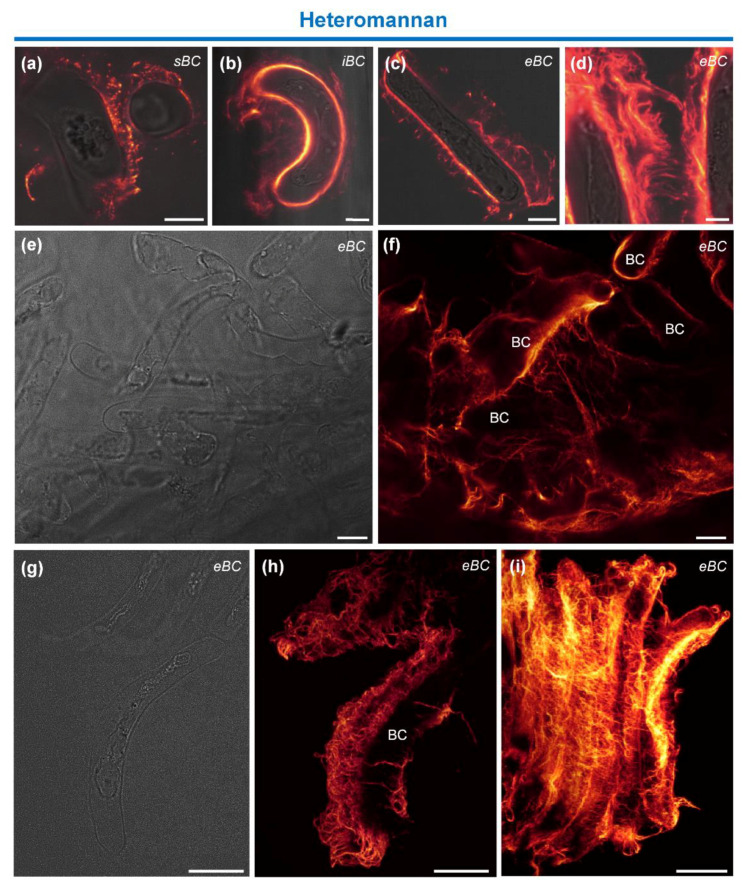
Immunolocalization of heteromannan epitopes. (**a**–**d**,**f**,**h**,**i**) Immunofluorescence images showing labeling of the cell wall and extracellular material in all BC morphotypes with the mAbs LM21. Stained fibrillary material is observed around the cells and between them. (**h**,**i**) Stack images reconstitution reveals a very dense hairy network covering the cells and holding several of them together. (**e**,**g**) Bright field images corresponding to fluorescence images in (**f**,**h**). sBC, spherical border cells; iBC, intermediate border cells; eBC, elongated border cells. Scale bars: (**a**–**c**) 10 µm; (**d**) 5 µm; and (**e**–**i**) 25 µm.

The presence of β-linked heteromannan was also investigated using the mAb LM21, which recognizes [β(1-4)-manno-oligosaccharides]2/5 [50]. As illustrated in Figure 6, the antibody labeled the cell wall either entirely or only partially (Figure 6a–c). In contrast, the extracellular material was densely labeled for all BC morphotypes and the fluorescent labeling always exhibited a fibrillar pattern (Figure 6d,f). As for XyG, immunostained heteromannans were observed as strands peeling off the surface of the cells into the extracellular mucilage (Figure 6f,h,i).

To further examine the fibrous structure containing XyG and heteromannan, we applied fluorescence signal reconstitution and deconvolution techniques. As illustrated in the 2D deconvolution images shown in Figure 5f and Figure 6h,i, the data reveal the presence of very dense filamentous and hairy-like networks that covered the cells of all BC morphotypes and held them together. Connections between the cells were also clearly visible.

#### 3.4.2. Staining of Cellulose

**Figure 7:**
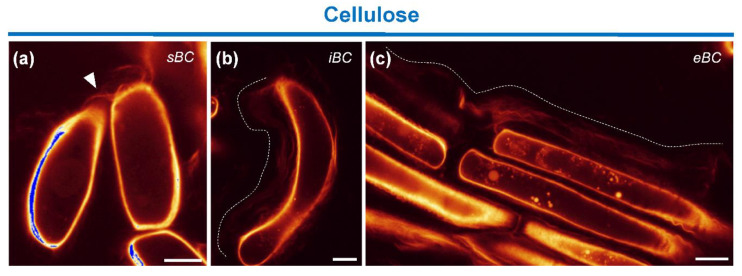
Staining of crystalline cellulose with the probe Direct Red 23. (**a**–**c**) Fluorescence images showing a strong staining of the cell wall of all BC morphotypes. A fibrillary material is also seen peeling off the cell surface into the extracellular space outside the cells ((**b**,**c**) white dote line). Some cells are interconnected via fluorescently stained material ((**a**) white arrowhead). sBC, spherical border cells; iBC, intermediate border cells; eBC, elongated border cells. Scale bar: 10 µm.

We stained cellulose using the fluorescent dye Direct Red 23, which is highly specific for this cell wall polysaccharide [51]. As shown in Figure 7, the dye strongly stained the cell walls of all BC morphotypes. In addition, fluorescence signal was observed outside the cells. Interestingly, a fibrous Direct Red 23-staining material was clearly seen peeling off the cell surface into the extracellular space (Figure 7a–c), suggesting that cellulose microfibrils can also be released into the mucilage surrounding BCs. Fluorescently stained cross-links between cells were also observed (Figure 7a).

#### 3.4.3. Immunolocalization of Pectin and Hydroxyproline-Rich Glycoprotein Epitopes.

We used two antibodies to examine the distribution of pectic polysaccharides, namely the mAb LM6 which recognizes [α(1–5) highly branched arabinan]5/6 of rhamnogalacturonan-I (RG-I) and the mAb LM8 which specifically binds to xylogalacturonan (XGA) [52,53,54].

**Figure 8:**
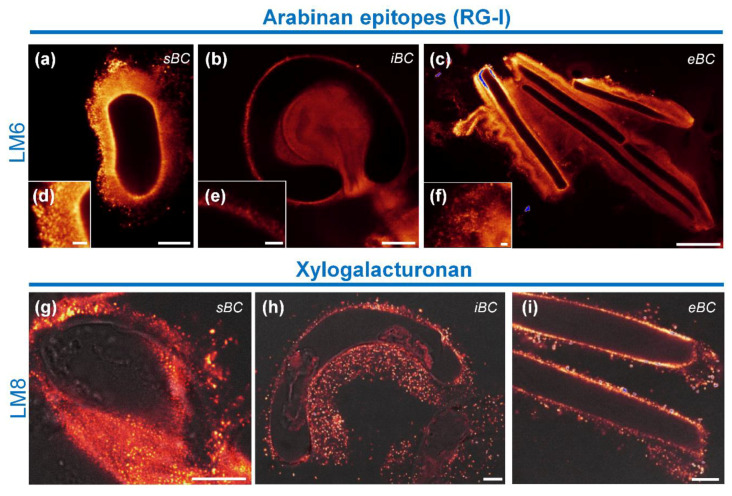
Immunolocalization of pectic epitopes. (**a**–**c**) Fluorescence images showing labeling with the mAb LM6 specific for highly branched arabinan chains of RG-I. Cell wall and secreted mucilage are strongly stained with the antibody. (**d**–**f**) Higher magnification images of the fluorescent signal highlighting its uniform punctate pattern. (**g**–**i**) Fluorescence images showing labeling with the mAb LM8 specific for XGA. A punctate staining is observed over the mucilage for the three morphotypes. Cell walls are also labeled in iBC and eBC, but much less in sBC. (**b**,**f**) Note that labeling of the secreted mucilage with both antibodies is polarized in iBC morphotypes, being mostly associated with the curved face of the cells. sBC, spherical border cells; iBC, intermediate border cells; eBC, elongated border cells. Scale bars: (**a**) 25 µm; (**b**,**g**–**i**) 10 µm; (**c**) 50 µm; and (**d**–**f**) 2.5 µm.

The mAb LM6 stained BC walls of all three BC morphotypes (Figure 8a–c). The antibody also stained the secreted mucilage surrounding the cells quite strongly. Interestingly, while labeling was observed evenly surrounding the entire sBC and eBC (Figure 8a,c), the signal exhibited a polarized distribution in isolated iBC, with the majority of the label being associated with the curved face of the cells (Figure 8b). Examination of fluorescent signal at a higher magnification showed a homogeneous punctate pattern of mucilage staining (i.e., numerous small fluorescence spots throughout the secreted mucilage) (Figure 8d–f).

Immunolabeling with the mAb LM8 was also observed over the cell walls of iBC and eBC (Figure 8h,i), but was rarely associated with the cell walls of sBC (Figure 8g). In contrast, the extracellular material was always labeled, although more or less abundantly, depending on the cell type. Labeling also showed a punctate fluorescence pattern that was less dense compared to the mAb LM6 labeling. Interestingly and as observed with the mAb LM6, the fluorescence signal associated with secreted mucilage was also found predominantly on the curved face for iBC, suggesting a polarized secretion of polysaccharide(s) containing epitopes recognized by LM6 and LM8 antibodies (Figure 8b,h).

In addition to pectins and hemicelluloses, we investigated the occurrence of arabinogalactan protein (AGP) and extensin epitopes in the RET (i.e., mucilage and BCs) using LM2 and JIM12 mAbs [55,56,57]. Our data show that both antibodies labeled the extracellular space surrounding the cells, suggesting the presence of these glycoproteins in the mucilage material (small spots or aggregates in Figure S5). The LM2 labeling was far less abundant in the mucilage surrounding eBC as compared to sBC and iBC (Figure S5a–c). As compared to the LM2, labeling with the mAb JIM12 was more diffuse and surrounding all BC morphotypes (Figure S5d–f). In contrast and surprisingly, the cell walls of all cell types were rarely labeled with either of these two mAbs.

Table S1 summarizes the data of cell wall glycan labeling and staining obtained for the different BC morphotypes.

#### 3.4.4. Immunolocalization of Histone H4 Protein and Staining of exDNA

**Figure 9:**
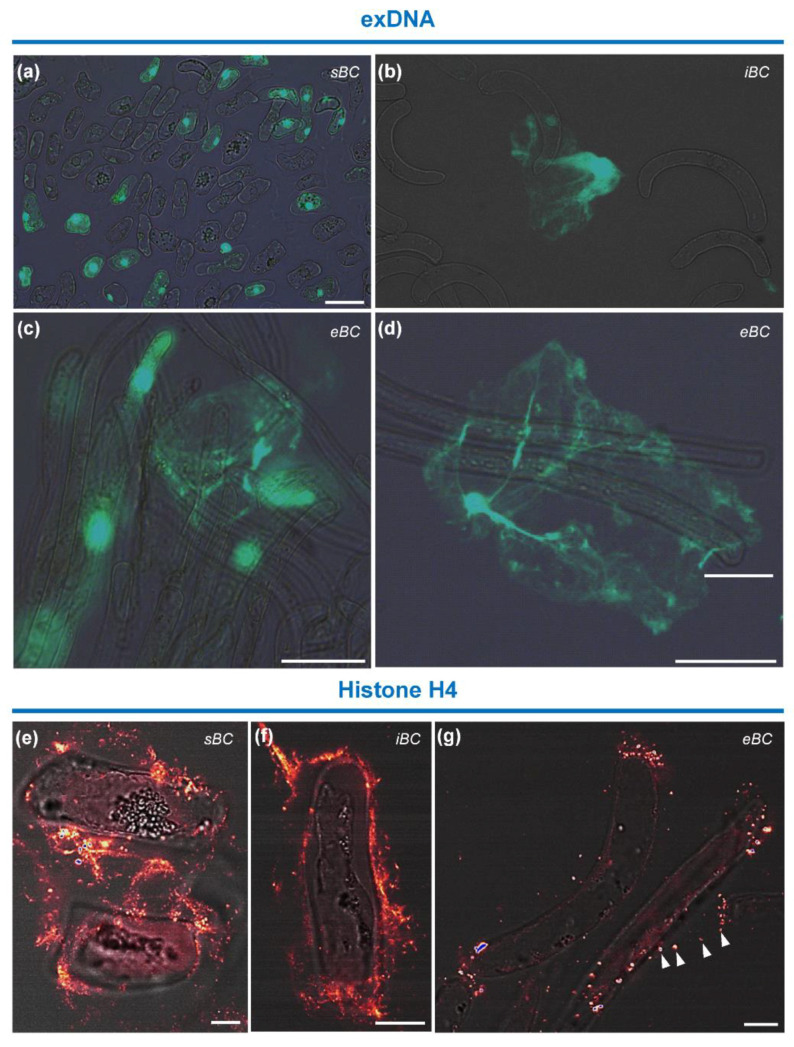
Staining of extracellular DNA and immunolabeling of histone H4 protein. (**a**–**d**) Images showing fluorescent staining of exDNA with the probe Sytox green. A fluorescence signal is observed in the extracellular matrix surrounding (**b**) iBC and (**c**,**d**) eBC. (**a**) No staining inside sBC. (**a**,**c**) Dead sBC or eBC are also stained. (**e**–**g**) Images showing labeling of the extracellular material surrounding the cells with anti-histone H4 antibody. Fluorescent spots can be seen along the filamentous material outside the cells (see arrowheads in (**g**)). Scale bars: (**a**–**d**) 50 µm; and (**e**,**f**) 10 µm.

One of the distinctive features of the RET is the presence of exDNA [15,16]. Here, we examined the presence of this component in soybean root BC and mucilage secretions using the DNA specific probe Sytox green. A clear fluorescence signal was observed in the extracellular material surrounding iBC and eBC (Figure 9b–d), but only rarely associated with sBC (Figure 9a). These data indicate that exDNA is released into the extracellular mucilage produced by soybean root BCs, most likely by iBC and eBC morphotypes.

Labeling of cells with the anti-histone H4 antibody showed a punctate signal in the extracellular area surrounding all three BC morphotypes (Figure 9e–g). The signal was sometimes visible along the filamentous structures between the cells (Figure 9e,f). In addition, in isolated eBC, fluorescence spots were observed to aggregate predominantly at the cell tips (Figure 9g). Therefore, histone 4 protein, which is known to bind DNA, is also present in mucilage secretions of soybean root BC, as previously reported for pea [25].

Remarkably, double immunolabeling of mucilage with anti-XyG and anti-histone H4 antibodies showed that the epitopes co-localized (see Figure S6). The fluorescent spot signals of histone H4 appeared to “run” over the XyG-labeled threads along the cell surface or outside the cells (Figure S6i).

### 3.5. Interaction of Root Tip with the Pathogen Oomycete Phytophthora parasitica (Breda de Haan)

**Figure 10:**
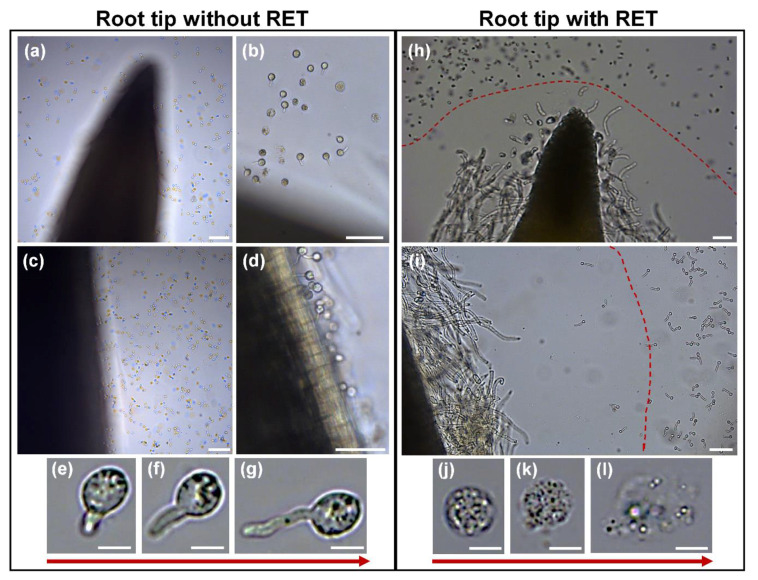
Interaction of root tip with the pathogen *Phytophthora parasitica*, effect of root extracellular trap (RET). (**a**–**d**) Root tips devoid of their border cells and mucilage or (**h**,**i**) intact root tips were inoculated with zoospores and observed after 15–30 min. (**e**–**g**) RET-less root tip was rapidly colonized by the zoospores that started to germinate after only 15 min. (**h**,**i**) In contrast, the zoospores did not or very rarely reached intact root tips (even after 30 min of inoculation) and did not colonize them. The vast majority of zoospores could not penetrate the zone of mucilage (red dashed line). (**j**–**l**) The very few zoospores that reached that zone or those that penetrated the mucilage underwent a rapid lysis and died 15–30 min after inoculation. Scale bars: (**a**,**c**) 100 µm; (**b**,**d**,**h**,**i**) 50 µm; and (**e**–**g**,**j**–**l**) 10 µm.

We investigated the impact of RET on the colonization of root tips by the oomycete *Phytophthora parasitica* (Breda de Haan). This soil-borne pathogen has been shown to infect a wide range of plant species. We inoculated root tips with zoospores and monitored the colonization process for the following 30 min (i.e., to monitor early colonization). Inoculation of zoospores was performed on root tips either devoid of RETs (i.e., the RET was carefully removed from the root tip) or still containing their RETs (intact root tips). In RET-less root tips, zoospores were present at the root apex (Figure 10a,b) and around the elongation zone (Figure 10c,d) after only 15 min of inoculation. A high number of zoospores were in contact with root cells and most of the zoospores started to germinate (Figure 10e–g). In intact root tips (in which the RET was not removed), nearly all the zoospores remained at a distance from the RET layer and could neither penetrate this network to reach the root cap nor reach the cell division or elongation regions of the root body (Figure 10h,i and video S1). Most of the zoospores were seen moving around the root apex without crossing the RET “borders” (Figure 10h,i and video S1). During this process, very few zoospores could reach the zone of contact with the RET and sometimes crossed “the borders” and penetrated the RET network. Careful observation of these zoospores showed that they rapidly burst and died leaking their cytoplasmic content into the surrounding environment (i.e., cell lysis) (Figure 10j–l and see video S1). In contrast, zoospores that remained distant from the zone of contact with RET displayed normal morphology and were able to initiate germination. Together, these data support a role of soybean RET in root protection during the early stages of infection by the pathogenic oomycete *P. parasitica*.

## 4. Discussion

BC release by root caps is an important biological process that regulates root development and protection [8,9,58]. In most species, this process is accompanied by the release of mucilage containing a variety of compounds that serve as a biological interface between the root and the rhizosphere and is essential for controlling biotic and abiotic interactions [1,59,60]. Although root BCs were reported in many species, thus far the Leguminosae species *Pisum sativum* L. remains the most studied species with regards to BC structure and function in defense [17,26].

Herein, we provide the first detailed characterization of root BCs and their secretions in *Glycine max*, an important crop member of the Leguminosae family. We show that soybean root tips release three BC morphotypes that all secrete substantial amounts of mucilage consisting predominantly of cell wall polysaccharides including heteromannan, a component that has not been reported previously in root secretions of any species so far. Our findings also show that: (i) exDNA and histone H4 protein, both involved in defense [24,28], were also present in the mucilage; and (ii) BCs and secretions were able to prevent *P. parasitica* zoospores from colonizing the root tip supporting their role in root protection.

### 4.1. Glycine Max (Merr) L. Root Tip Releases Different Cell Morphotypes Surrounded by Mucilage

Our observations provide additional evidence that different root BC morphotypes are released depending on the plant species [61,62,63]. Similar to previous observations for pea seedlings [19], we found that soybean root tips release distinct populations of BCs. We classified the cells into three morphotypes based on their size and morphology as spherical (sBC), intermediate (iBC) and elongated (eBC) BCs. However, in contrast to pea where all BCs were released as individual cells fully separated from each other [19], in soybean, the eBC were released in the form of a stack of several firmly attached cells (see Figure 2). Remarkably, very large stacks of eBC were observed to be produced by root tips of *Acacia mangium* (Wild.) [11,63]. The authors described these cell structures as a novel type of BLCs arranged in a brick-wall pattern, forming a large “fibrous tissue” floating around the root apex. This tissue covered a large part of the root apex and was observed to peel off from the epidermis in the elongation zone [11] (see Figure 2g,h). The authors suggested that the tissue provided physical protection to the root during growth by reducing friction between the root and soil particles thereby facilitating root growth. In soybean root, the cell stacks were consistently observed in the root elongation region and were relatively smaller than the acacia fibrous tissue. Nonetheless, intercellular attachment of cells in both tissues is strong and supports its role in root protection against pathogen penetration and toxic metals [11]. In relation to metal toxicity, Cai et al. [34,35] showed that BCs and their mucilage are involved in protecting the root against Al-toxicity in an Al-tolerant cultivar of soybean. The authors clearly showed that Al-treatment caused an enhanced mucilage secretion and that removal of BCs significantly increased Al-induced inhibition of root growth.

In this context, it is worth noting that all the cells produced by the soybean root tips were surrounded by mucilage of significantly different densities and abundance (see Figure 4) and clearly contained pectic polysaccharides, including RG-I and xylogalacturonan (see Figure 8). Remarkably, the iBC curved cells (some of which had a circular shape) exhibited polarized polymer secretion with nearly all of the labeling being associated with the mucilage localized to the shorter side of the cell. Mravec et al. [18], who suggested that the composition of the cell wall is different in the curvature zone to initiate BCs detachment, also observed such a polarized secretion in pea root cells. However, the functional significance of this asymmetric distribution of mucilage is currently unknown.

### 4.2. Heteromannan and XyG Secreted by BCs Maintain Mucilage Architecture

One of the most novel observations we made is the occurrence of heteromannan and XyG networks in the cell wall and secreted mucilage surrounding soybean BCs. Interestingly, in spermatophytes, mannan and glucomannan are generally less abundant in primary cell walls than other hemicelluloses (e.g., XyG). They are sometimes described as being predominantly associated with secondary cell walls and are believed to fulfill a structural role [64,65]. Furthermore, heteromannan polysaccharides have never been previously described in root secretions of any plant species including pea, whereas XyG has been reported in root exudates of several plants, including maize, wheat, pea and Arabidopsis [20,66,67].

The immunolabeling pattern of the hemicellulosic polysaccharide XyG in the mucilage (see Figure 5 and Figure 6), appeared as a fibrous network peeling off the cell surfaces, surrounding the cells and holding them together. The presence of bridges connecting the cells at their apical edges was also observed. Interestingly, labeling of heteromannan was even stronger in the mucilage than that of XyG. The mAb LM21 used in this study has been described to bind equally to mannan, glucomannan and galactoglucomannan [50]. The labeling pattern was slightly different compared to that of the XyG network, revealing a very dense fibrillary network completely coating the cells.

Based on our observations and the known functions of these polysaccharides, we speculate that XyG and heteromannan are two critical structural components that form a structural scaffold required for mucilage integrity and cohesion surrounding BCs. Additionally, this scaffold would provide a template on which other components of the mucilage, including anti-microbial molecules, would assemble for the overall organization and functioning of the RET.

Whether XyG and heteromannan interact with each other and with other components of the mucilage network is not known. However, XyG is known to cross-link cellulose via hydrogen bonds in planta [68,69] and associations between cellulose microfibrils and mannan polymers were reported to form in the cell wall [70]. XyG is also able to interact with pectic polysaccharide RG-I [71,72], but interactions between mannan polymers and pectins have not been described so far. Interestingly, heteromannan polysaccharides are commonly found in the seed mucilage of many species [73,74,75]. In Arabidopsis, these components were shown to play a central function in mucilage architecture by stabilizing the cellulose rays and maintaining adherence of pectic polysaccharides to the seed surface [74]. Mutants deficient in heteromannan synthesis, such as muci10 for instance, were shown to exhibit major alterations in seed mucilage density and structure [75,76].

Therefore, interactions of heteromannan and XyG with other polysaccharides such as cellulose and pectin might occur, at least in certain regions in RET mucilage. These associations would strengthen the mucilage network and the cross-links present between BCs themselves. In our SEM images of root tips (Figure 1), fibrillary structures which may correspond to the scaffold formed by the hemicellulosic polysaccharides, XyG and heteromannan, were clearly seen surrounding BCs.

### 4.3. The RET: A Protective Physical and Chemical Barrier?

We previously defined the RET as an association of root BCs and their mucilage secretions [15]. RET network was shown to contain a mixture of components including polysaccharides, proteoglycans, exDNA, histones and various anti-microbial molecules involved in the control of root interactions with soil borne microbes [14,15,16,19,77].

Our data show that the soybean RET prevented *Phytophthora* zoospores from reaching and colonizing root tissues. It also affected their survival. Generally, zoospores could not penetrate the RET layer possibly due to its shield-like function, but, when some of them do, they burst and die. As discussed above, the RET network would provide a protective physical barricade as well as a matrix supporting the assembly of anti-microbial molecules [15]. XyG and heteromannan are highly abundant in soybean RET and are known as structural polymers able to form a stable rigid network by themselves or in association with other molecules (e.g., cellulose) [78,79,80]. We believe that these components would interact directly or indirectly with other polysaccharides and/or glycoproteins of the RET (e.g., RG-I and extensin) to enhance mechanical properties of mucilage and reinforce RET function as a physical barrier against pathogen invasion.

It is relevant to note that, during infection of pea roots with the soil borne fungus *Nectria hematoccoca* (Berk. & Broome), BCs and secretions bound the pathogen hyphae forming a mantle that could detach entirely from the root tip leaving it free of infection [27]. In this interaction, the invading fungus was kept away from the root tip much like *Phytophthora* zoospores in our observations and fungal hyphae did not penetrate any BC. The study [27] also showed that removal of BCs from root before inoculation with fungal spores resulted in an increase in root tip infection

In addition to its structural properties, the soybean RET acts as an anti-microbial chemical complex likely to be responsible for the observed effect on zoospores (e.g., cell lysis and death). Soybean RETs contain exDNA and histones, both of which were shown to inhibit pathogen growth and induce microbial killing in plant and animal systems [24,25,81]. In mammals, exDNA of neutrophil traps was shown to have a direct bactericidal function by disrupting cell membranes of pathogenic bacteria causing their death [81]. It was also shown that exDNA, in conjunction with histone H4 released by pea RET, had a toxic effect on the soil borne pathogen *Ralstonia solancerum* [24].

Therefore, it is reasonable to suggest that the RET-induced killing of *Phytophthora p.* zoospores may be due to the combined effects of histones and exDNA also found in soybean RETs (possibly originating from dead BC). Nonetheless, other anti-microbial molecules might also occur in soybean RET and could contribute to the killing of the zoospores. In addition to exDNA and histones, diversity molecules able to trap, inhibit or kill microbial pathogens were reported in root secretions and in neutrophil extracellular traps of mammals including proteins, peptides, phytoalexins, enzymes, ROS, etc. [14]. However, such molecules are currently not identified in soybean root secretions and will await future investigations.

## 5. Conclusions

Our findings reveal that root BCs of soybean release mucilage secretions enriched in heteromannan and XyG that form a dense fibrillary network likely to play a role in maintaining the structural integrity of the RET. The mucilage also contains exDNA and histone H4, two components known to have a defensive function in plants and mammals [14,24]. Our findings also support the role of root BCs and secretions in soybean root protection against the pathogen *Phytophtora parasitica*.

## Supplementary Materials

The following are available online at https://www.mdpi.com/2073-4409/9/10/2215/s1, Figure S1: Cell viability and negative control. Figure S2: Visualization of mucilage in curved iBCs using India ink staining. Figure S3: Immunolabeling negative controls. Figure S4: Immunolocalization of xyloglucan epitopes. Figure S5: Immunolabeling of hydroxyproline-rich glycoproteins epitopes with anti-arabinogalactan protein (LM2) and anti-extensin (JIM12) antibodies. Figure S6: Double immunofluorescence labeling of xyloglucan and histone H4 in secreted mucilage. Table S1: Summary of the glycan immunolabeling and staining data obtained in the present study. Table S2: Cell size measurements (length versus width) and percent of viable versus non-viable cells. Video S1: Interaction of soybean root tip with the pathogen *Phytophthora parasitica* (Breda de Haan) and the effect of root extracellular trap (RET).
