# Supplementary figures and images for: Root Border Cells and Mucilage Secretions of Soybean, Glycine Max (Merr) L.: Characterization and Role in Interactions with the Oomycete Phytophthora Parasitica
Source: Cells. 2020 Sep 30;9(10):2215. doi: 10.3390/cells9102215 (PMC7650559; doi:10.3390/cells9102215)

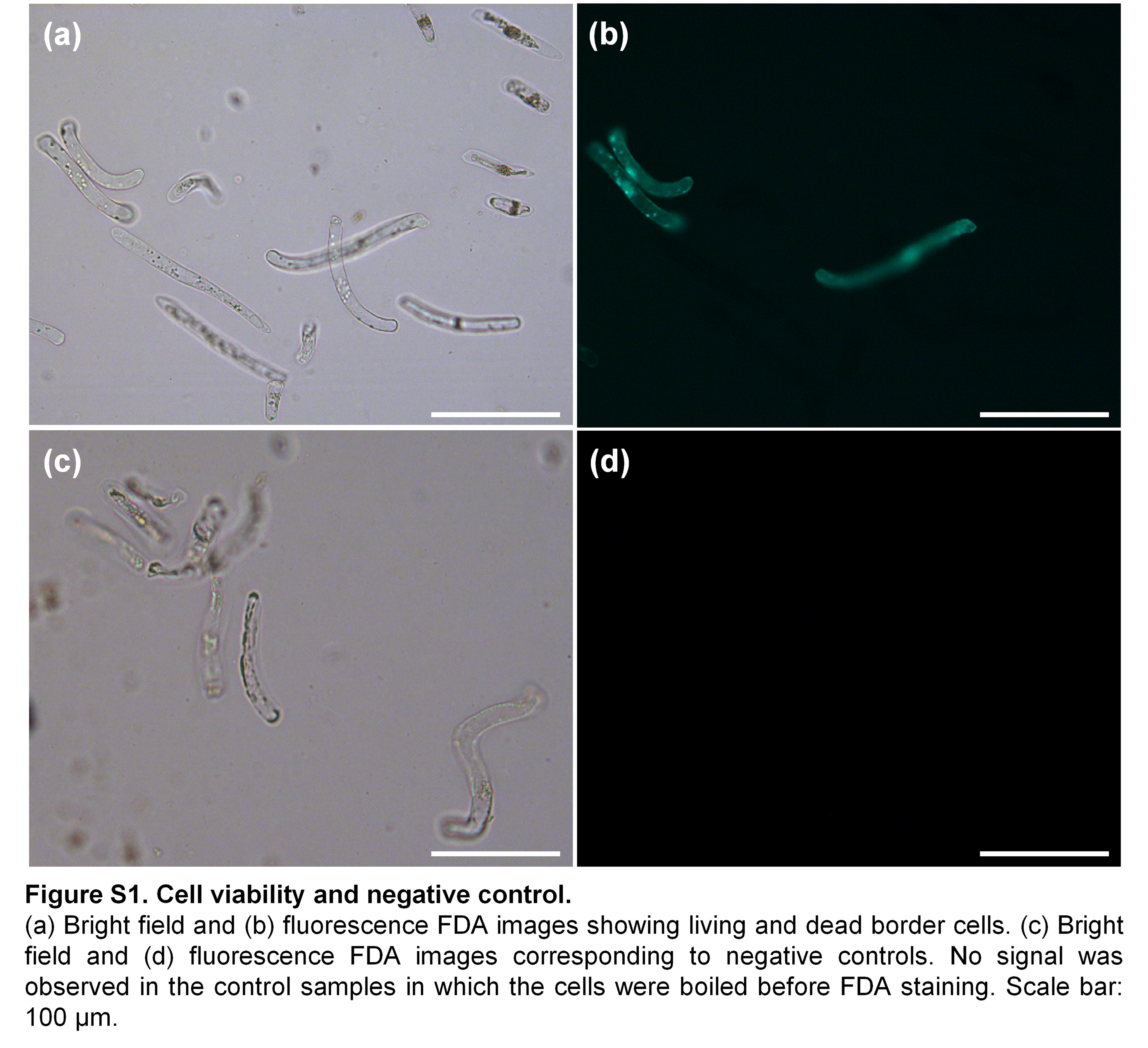

Supplement: Supplementary file 1 [file cells-09-02215-s001.zip › Supplementary files cells-937886 revised/Figure S1.tif]

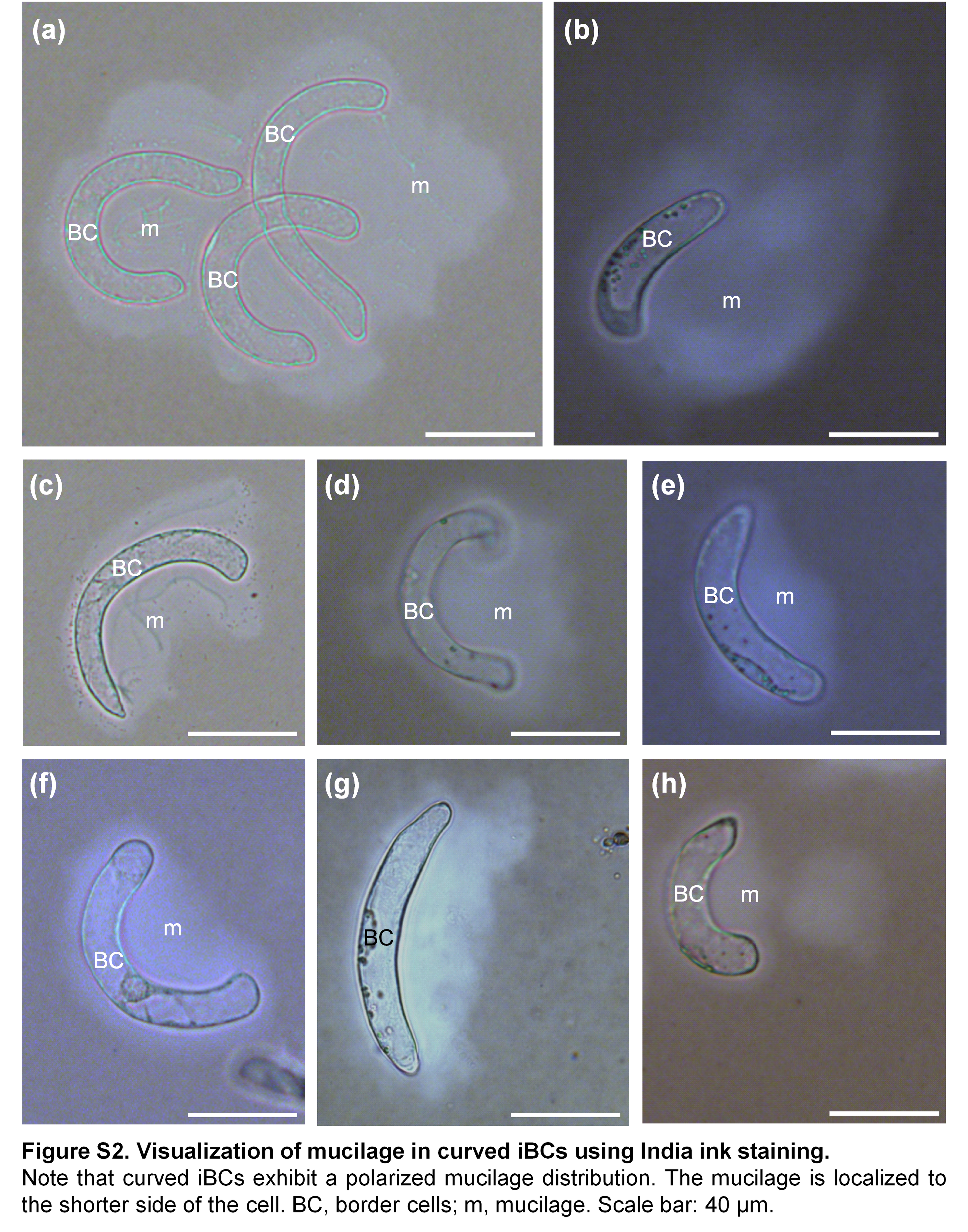

Supplement: Supplementary file 1 [file cells-09-02215-s001.zip › Supplementary files cells-937886 revised/Figure S2.tif]

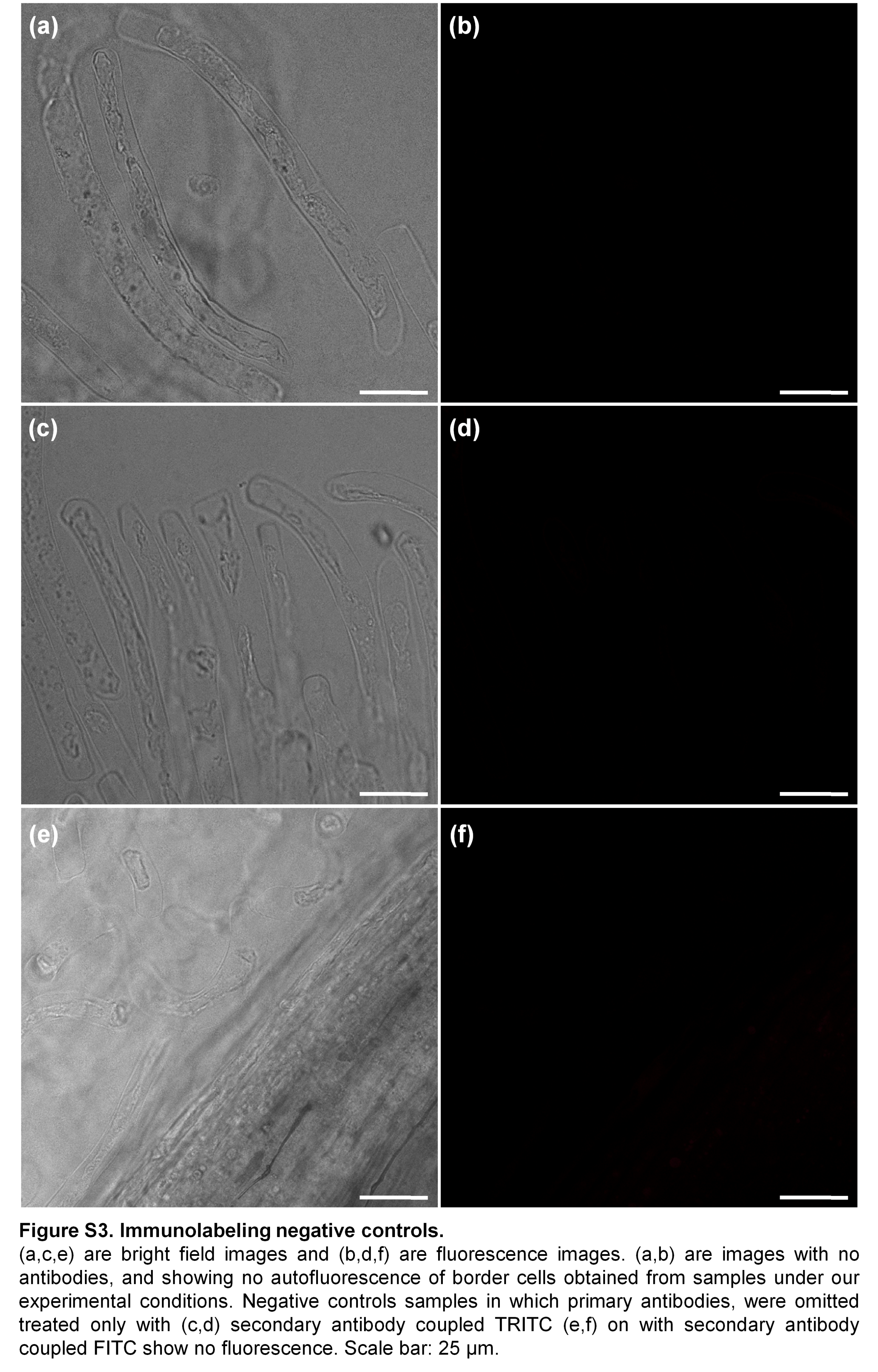

Supplement: Supplementary file 1 [file cells-09-02215-s001.zip › Supplementary files cells-937886 revised/Figure S3.tif]

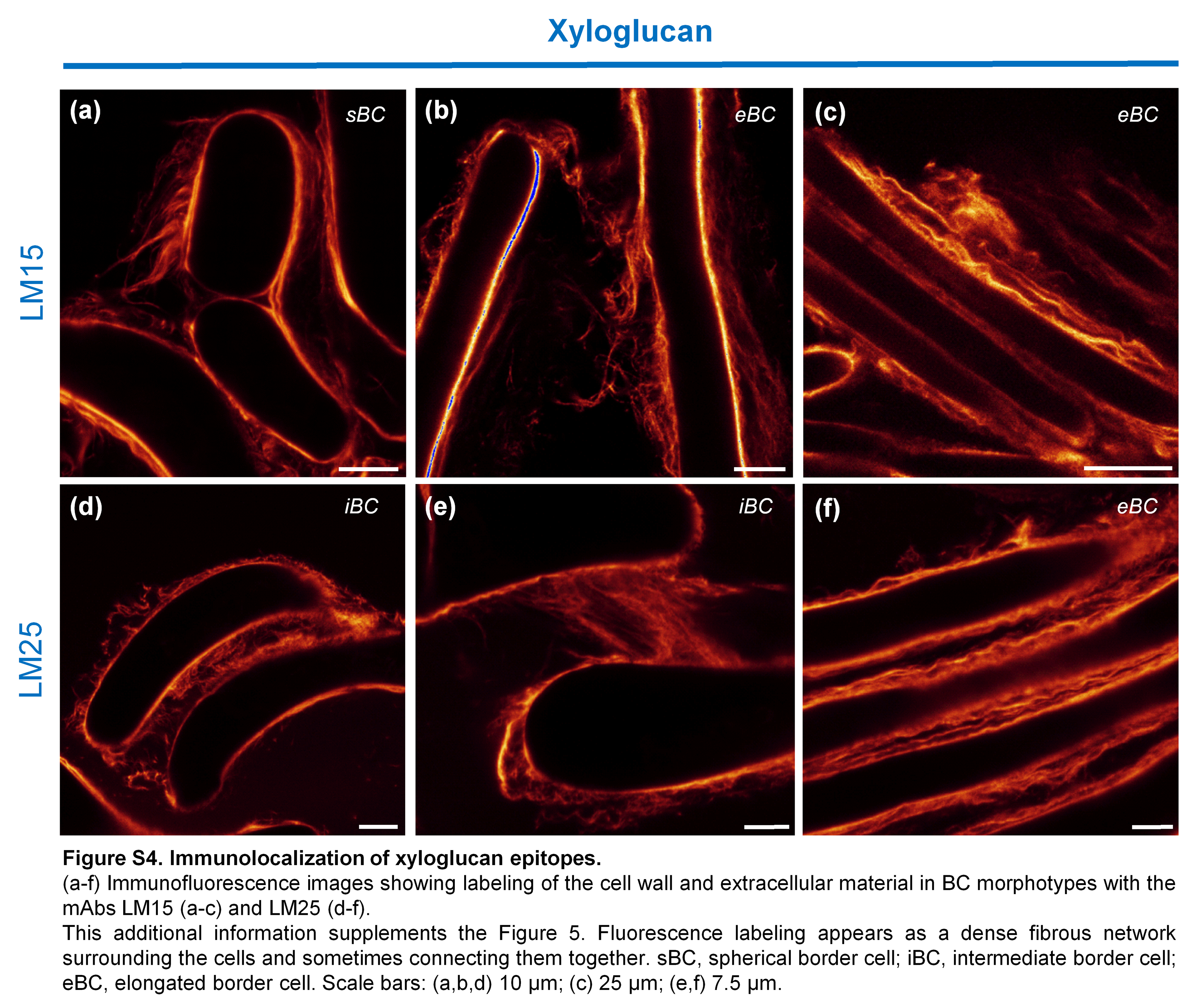

Supplement: Supplementary file 1 [file cells-09-02215-s001.zip › Supplementary files cells-937886 revised/Figure S4.tif]

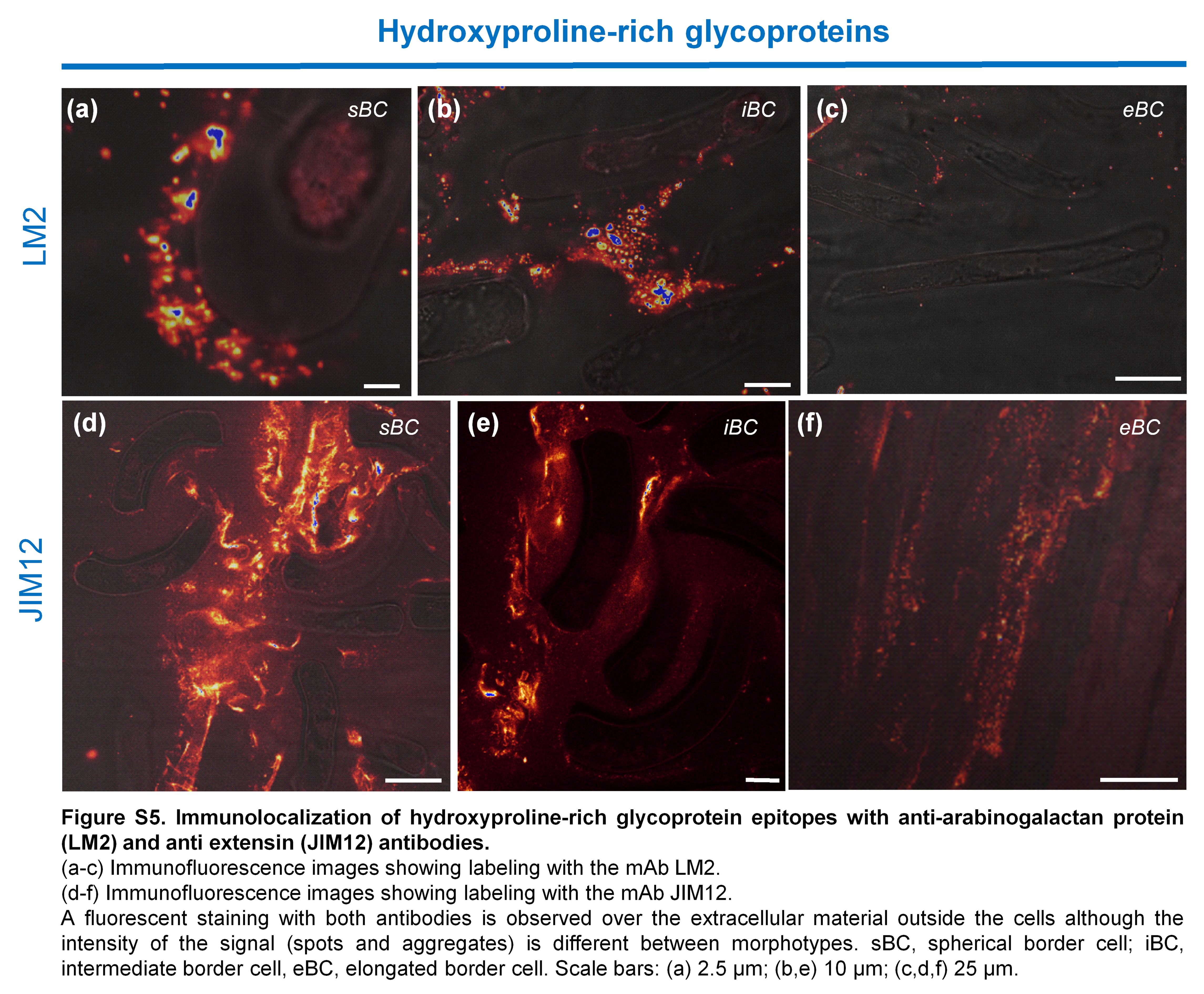

Supplement: Supplementary file 1 [file cells-09-02215-s001.zip › Supplementary files cells-937886 revised/Figure S5.tif]

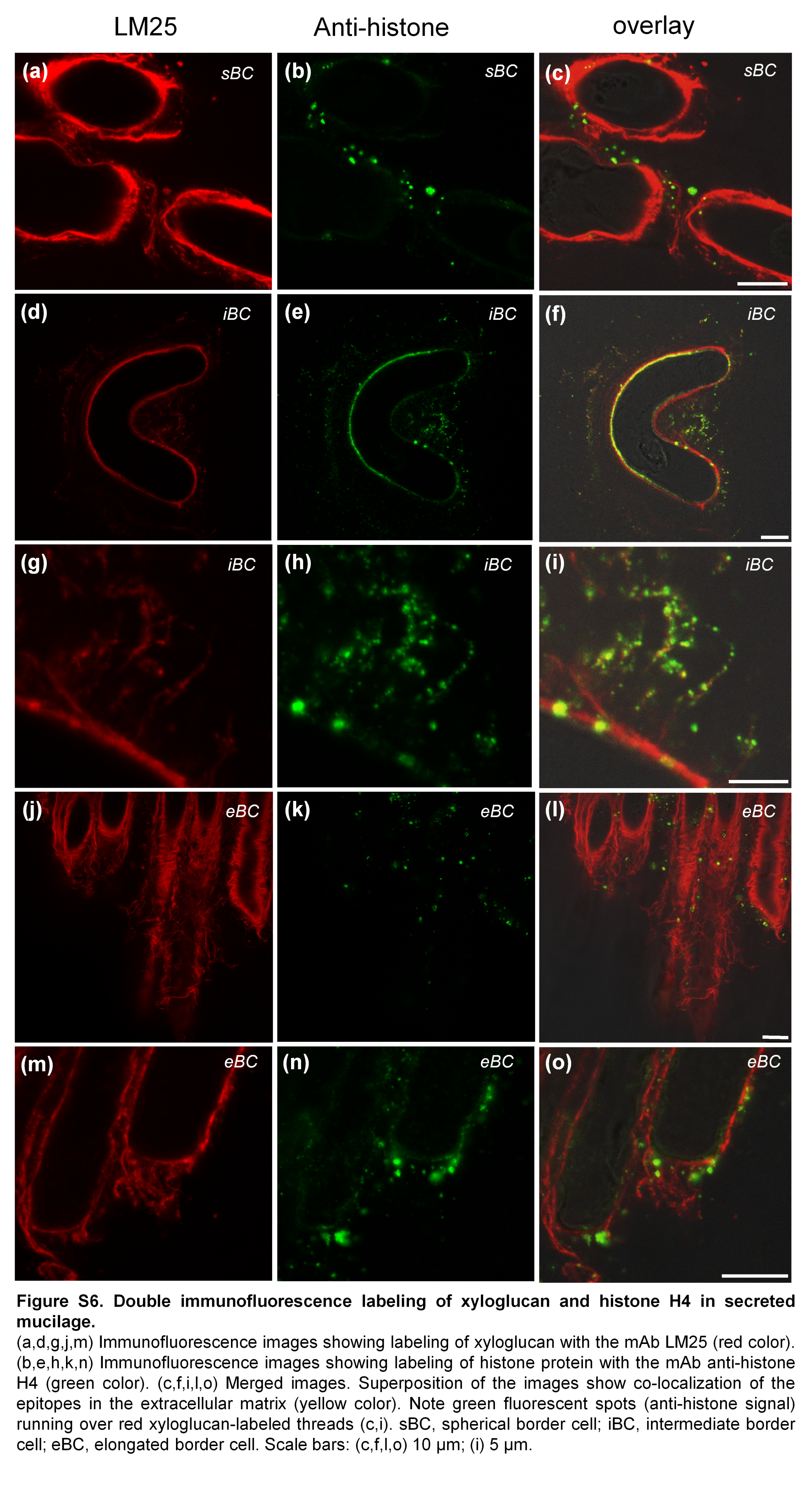

Supplement: Supplementary file 1 [file cells-09-02215-s001.zip › Supplementary files cells-937886 revised/Figure S6.tif]
